# A Protein-Based Hydrogel for *In Vitro* Expansion of Mesenchymal Stem Cells

**DOI:** 10.1371/journal.pone.0075727

**Published:** 2013-09-19

**Authors:** Jingyu Wang, Jinxiu Zhang, Xiaoli Zhang, Hao Zhou

**Affiliations:** 1 State Key Laboratory of Medicinal Chemical Biology, Nankai University, Tianjin, China; 2 College of Life Sciences, Nankai University, Tianjin, China; 3 College of Life Science, Hebei Normal University, Shijiazhuang, Hebei, China; Instituto Butantan, Brazil

## Abstract

Hydrogels are widely used as scaffolds in tissue engineering because they can provide excellent environments for bioactive components including growth factors and cells. We reported in this study on a physical hydrogel formed by a specific protein-peptide interaction, which could be used for the three dimensional (3D) cell culture of murine mesenchymal stem cells (mMSC). The mMSC kept dividing during the 7-day culture period and the metabolic-active cell number at day 7 was 359% more than that at day 1. This kind of physical hydrogel could be converted to a homogeneous solution by firstly adding an equal volume of culture medium and then pipeting for several times. Therefore, mMSC post culture could be easily separated from cell-gel constructs. We believed that the protein-based hydrogel system in this study could be developed into a promising scaffold for *in vitro* expansion of stem cells and cell therapy. This work would be in the general interests of researchers in the fields of biomaterials and supramolecular chemistry.

## Introduction

The generation of genetically encoded protein-based hydrogels for an array of applications, such as tissue regeneration, 3D cell culture and drug delivey, has been much anticipated[1], [2], [3], [4], [5], [6]. 3D cell culture is evolving rapidly. Different from traditional 2D culture, its continuous sophistication makes us understanding of cellular microenvironment well and guides us comprehending how the basic building blocks of biological systems are integrated into the dynamic landscape of tissue physiology[7], [8], [9]. In order to form protein-based hydrogels, genetically encoded proteins with multiple cross-linking sites are needed[10]. These proteins can then be used to cross-link polymers or proteins via covalent[11], [12], [13], [14], [15], [16], [17], [18], [19] or non-covalent[20], [21], [22], [23], [24] interactions, leading to hydrogels formation. Among the non-covalent interactions, specific interactions between proteins and their cognate peptide lingands have attracted recent research interests. For example, hydrogels formed by a specific protein-peptide interaction can respond to external stimuli such as ionic strength change[22] and calcium addition[4]. Therefore, they hold big potential for controlled drug delivery, cells encapsulation, and cells delivery[20], [21], [25], [26], [27], [28], [29].

In order to produce protein-based hydrogels *via* specific protein-peptide interactions, obtaining proteins with multiple binding sites to their peptide ligands is the key. There are two strategies to obtain these kinds of proteins. One strategy is designing and producing recombinant proteins with tandem repeating binding domains[22], [23], [30], and the other one is making recombinant proteins that can form oligomeric structures[21], [24]. Until now, there are only several proteins designed by these two strategies which been used for the formation of protein-based hydrogels.

Recently, Yang *et al.* reported a tetrameric recombinant protein (ULD-TIP-1)-based hydrogel[24]. In their study, the addition of ULD-TIP-1 enhances the interactions between fibers which have been formed by the peptide of Nap-GFFYGGGWRESAI. In this study, we used the tetrameric recombinant protein (ULD-TIP-1) to cross-linker four-armed PEG through a specific protein-peptide interaction, thus resulting in the hydrogel formation. We also demonstrated that hydrogels in our study were well suitable for three dimensional (3D) expansion of mMSC.

## Materials and Methods

### Materials and general methods

Fmoc-amino acids were obtained from GL Biochem (Shanghai, China). Maleimide-end-capped four-armed poly(ethylene glycol) [company claim data: molecular weight (Mw) = 2.3×10^4^] was obtained from Laysan Bio (Arab, AL). The Live/Dead Viability/Cytotoxicity Kit was purchased from Invitrogen (Life Technologies, USA). Low glucose Dulbecco's Modified Eagle Medium with GlutaMAX was purchased from GIBCO (Life Technologies, USA). Murine mesenchymal stem cells (mMSC) specific fetal bovine serum (FBS) was purchased from Hyclone (Thermo Scientific, USA). Trypsin (0.25%)+EDTA and Penicillin/streptomycin were purchased from GIBCO (Life Technologies, USA). Cell Counting Kit-8 was purchased from Beyotime (Jiangsu, China). All the other Starting materials were obtained from Alfa Aesar (USA). Commercially available reagents were used without further purification, unless noted otherwise. Nanopure water was used for all experiments. All other chemicals were reagent grade or better.

The synthesized compound 1 (peptide of CGGGRGDWRESAI, Figure S1) and PEG/PEG-peptide were characterized by ^1^H NMR (Bruker ARX 300) using DMSO-d_6_ and H_2_O-d_2_ as the solvent, respectively (Figure S2, Figure S3). HPLC was conducted at LUMTECH HPLC (Germany) system using a C_18_ RP column with MeOH (0.1% of TFA) and water (0.1% of TFA) as the eluents. LC-MS was conducted at the LCMS-20AD (Shimadzu) system, and rheology was performed on an AR 2000ex (TA instrument) system using a parallel plates (40 mm) at the gap of 500 µm.

### Protein expression and purification

To make a single-chain fusion protein of the ULD-TIP-1 from human, DNA fragments corresponding to the ULD domain of human SATB1 (residues 71–172) and human TIP-1 were amplified by polymerase chain reaction (PCR). The single open reading frame was cloned into an in-house modified version of the pET32a (Novagen) in which the S-tag and the thrombin recognition site were replaced with a sequence encoding a PreScission protease-cleavable segment (Leu-Glu-Val-Leu-Phe-Gln-Gly-Pro). The resulting protein contained a Trx-His_6_-tag in its N-terminus.

BL21(DE3) CodonPlus *Escherichia coli* cells harboring the expression plasmid were grown in LB medium at 37°C until the OD_600_ reached 0.6 and then induced with 0.3 mM isopropyl-β-D-thiogalactoside at 16°C for about 16–18 h. After being spun at 5,000 r.p.m. for 15 min, *E. coli* cells were re-suspended in T_50_N_500_I_5_ buffer (50 mM Tris-HCl pH 7.9, 500 mM NaCl and 5 mM imidazole) supplemented with 1 mM phenylmethylsulfonyl fluoride, 1 µg/mL leupeptin and 1 µg/mL antipain. The cells were then lysed by AH-1500 (ATS Engineering Limited). After the lysates had been centrifuged at 18,000 r.p.m. for 30 min, the supernatant was loaded onto a Ni-NTA agarose column (Qiagen) that was equilibrated with T_50_N_500_I_5_ buffer. The Ni-NTA column was washed with 3 column volumes of T_50_N_500_I_5_ buffer. The Trx-his_6_-tagged protein was eluted with T_50_N_500_I_5_ buffer containing 500 mM imidazole. The eluted proteins loaded on a HighLoad 26/60 Superdex-200 size-exclusion column (GE Healthcare) and eluted with T_50_N_100_E_1_D_1_ buffer (50 mM Tris-HCl pH 8.0, 100 mM NaCl, 1 mM EDTA and 1 mM DTT) at a flow rate of 2.5 ml/min. Each fraction of the column elute was 5 ml. The protein peak was identified by SDS-PAGE gel. After digestion with PreScission Protease to cleave the N-terminal Trx-His_6_-tag, the target protein was purified on a Hiprep Q FF 16/10 anion-exchange column (GE Healthcare) by eluting with a linear gradient of NaCl up to 500 mM (10 column volumes). The final purification step was size-exclusion chromatography on a HiLoad 26/60 Superdex 200 column in 50 mM PBS pH 7.4, 100 mM NaCl and 1 mM EDTA[24]. The protein was concentrated to 40 mg/mL for preparation of hydrogel. The purified ULD-TIP1 protein was analyzed by 15% SDS-PAGE.

### Synthesis and characterizations

#### Peptide systhesis

The peptide derivative was prepared by solid phase peptide synthesis (SPPS) using 2-chlorotrityl chloride resin and the corresponding N-Fmoc protected amino acids with side chains properly protected by a tert-butyl group or Pbf group. The first amino acid was loaded on the resin at the C-terminal with the loading efficiency about 0.6 mmol/g. 20% piperidine in anhydrous N,N′-dimethylformamide (DMF) was used during deprotection of Fmoc group. Then the next Fmoc-protected amino acid was coupled to the free amino group using O-(Benzotriazol-1-yl)-N,N,N′,N′-tetramethyluroniumhexafluorophosphate (HBTU) as the coupling reagent. The growth of the peptide chain was according to the established Fmoc SPPS protocol. After the last amino acid coupling, excessive reagents were removed by a single DMF wash for 5 minutes (5 mL per gram of resin), followed by five steps of washing using DCM for 2 min (5 mL per gram of resin). The peptide derivative was cleaved using 95% of trifluoroacetic acid with 2.5% of TIS and 2.5% of H_2_O for 30 minutes. 20 mL per gram of resin of ice-cold diethylether was then added to cleavage reagent. The resulting precipitate was centrifuged for 10 min at 4°C at 10,000 rpm. Afterward the supernatant was decanted and the resulting solid was dissolved in DMSO for HPLC separation.

#### Preparation of PEG-peptide

We designed and synthesized a small molecular peptides (CGGGRGDGWRESAI). C could reacted with maleimidated PEG as it containing a thiol[18], with GGG as a linker, and WRESAI could tightly bind to TIP-1[31]. Peptides RGD could combine with integrin of the cell surface, which played a role in cell adhesion[32]. A mixture of cysteine-end-capped peptide (CGGGRGDGWRESAI) and maleimidated PEG (molar ratios of the peptide to maleimide group was 10∶1) in dimethyl formamide (DMF)was gently stirred overnight. Subsequently, this reaction product was dialyzed against filtrated H_2_O(dialysis membranes MWCO 8000∼14000) for 6 hours, repeating 4 times to remove unreacted peptide and DMF. Finally, it was freeze-dried to give a flaxen solid (PEG-peptide).

#### Preparation of hydrogel

20 mg/mL of PEG-peptide was dissolved in a PBS buffer solution (pH = 7.4) as a clear solution, an equal volume of PBS buffer solution (pH = 7.4) containing 40 mg/mL of ULD-TIP-1 was then added. The gel would form instantly. And the final concentration of PEG-peptide and protein in the gel was 1 wt% and 2 wt%, respectively.

#### Rheology

Rheological test was done on an AR 2000ex (TA instrument) system. 40 mm parallel plates were used during the experiment at the gap of 500 µm. For the dynamic time sweep, the solution of PEG-peptide was directly transferred to the rheometer and then equiv. of ULD-TIP-1 was added and it was conducted at the frequency of 10 rad/s and the strain of 1%. The gels were then characterized by the mode of dynamic strain sweep in the region of 0.1%−10% at the frenquency of 10%. Dynamic frequency sweep was performed in the region of 0.1–100 rad/s at the strain of 1%.

#### Scanning Electron Microscopy (SEM)

The silica wafer was cleaned with the assistance of sonication in ethanol for 10 minutes, and then a thin layer of the sample was cast on it and freeze-dried in a lyophilizer overnight. A layer of gold was spluttered on the sample by vacuum spray to make conduction surface. The SEM was done on a Hitachi X650 system (Japan) operating at 15 kV.

### Cell culture in hydrogel

mMSC substrate contain Low glucose Dulbecco's Modified Eagle Medium with GlutaMAX supplemented with 10% FBS (fetal bovine serum), 100 units/mL of penicillin, and 100 µg/mL streptomycin. Prior to 3D-culture, cells within a sub-confluent monolayer were trypsinized using trypsinase (0.25%)–EDTA (0.02%) solution and re-suspended in mMSC substrate (DMEM plus 10% FBS and 1% Penicillin/Streptomycin solution). Prior to preparation of the hydrogel, the powder (polymer) was weighed and sterilized by UV light for 60 min. 15 µL of mMSC cell suspension in mMSC substrate (DMEM plus 10% FBS and 1% Penicillin/Streptomycin solution) containing PEG-peptide and cells (2.0×10^6^ cells/ml hydrogel) was pipetted into each insert of the 96-well plate. 15 µL of the ULD-TIP-1 solution was then transferred to 96 well plates (30 µL of gel per well). After 0.5 hour incubation, 300 µL of mMSC substrate (DMEM plus 10% FBS and 1% Penicillin/Streptomycin solution) was added on the top of the hydrogel. The 96-well plate was maintained in a 37°C/5% CO_2_ incubator.

### Live–dead assay

Viability of encapsulated cells was tested by a Live–Dead assay (Sigma-Aldrich) performed 1day, 3days, 5days, and 7days post culture. The cell–gel constructs were washed three times with DMEM medium without FBS, and then a 60 µL aliquot of the assay solution containing 4 µM EthD-1 (ethidium homodimer-1) and 2 M calcein AM was pipetted onto each cell–gel construct. After 30 min incubation at room temperature, the constructs were observed using a Nikon Eclipse TE2000-U fluorescence microscope with excitation filters of 450–490 nm (green, Calcein AM) and 510–560 nm (red, EthE-1).

### Determination of cell proliferation rate by CCK-8

To quantify cell proliferation inside the cell-gel constructs, a CCK-8 assay was performed at a series of time points. A 3D Culture standard was made by encapsulating cells into hydrogels following the above 3D-culture procedure. To perform the CCK-8 assay, each cell-gel construct was incubated with 10 µL of CCK-8 agent in serum-free DMEM (300 µL). The plates were then incubated in the 5% CO_2_ incubator for 4 h at 37°C. The absorbance at 450 nm was determined using the microplate reader (MultiskaniMark, Bio-Rad, USA).

## Results and Discussion

**Figure 1:**
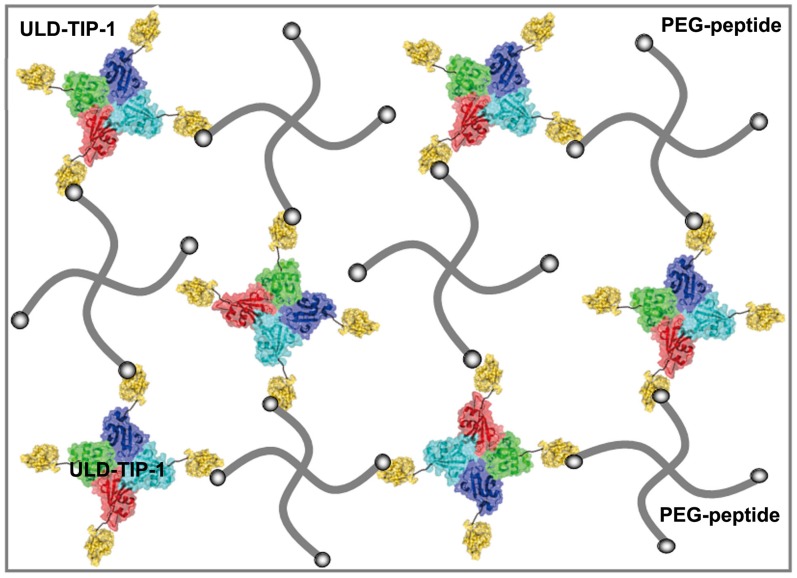
A cartoon representation to illustrate the formation of the hydrogels. 3D networks of the hydrogels are formed by the specific protein-peptide interaction. The blue, green, red and cyan represent ULD tetramer; the yellow represent TIP-1 protein; the grey thick line represent PEG-peptide; the grey balls represent hexapeptide of WRESAI which can bind with TIP-1.

Recently, Yang group had reported that a tetrameric recombinant protein of ULD-TIP-1 from mouse could increase cross-linking points of self-assembled nanofibers, thus leading to molecular hydrogelations[24]. We opted to develop hydrogels based on ULD-TIP-1 and polymers and test whether they were suitable for 3D cell culture or not. The ULD-TIP-1 had four peptide binding sites, which might be used to cross-link polymers for hydrogelations (Figure 1). Therefore, we expressed and purified ULD-TIP-1 (Figure S6) from human (only one residue difference to ULD-TIP-1 from mouse) to test whether it could form hydrogels or not in the presence of four armed PEG polymer terminated at peptide ligands.

### Rheological measurement and SEM

**Figure 2:**
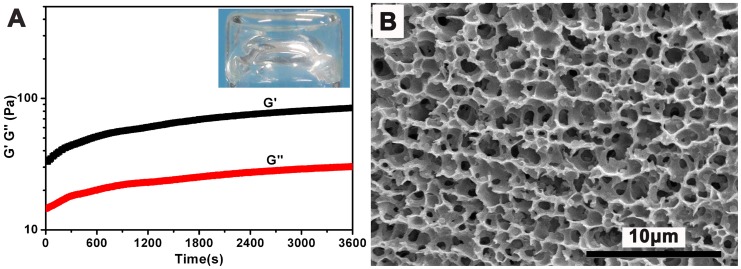
Rheological measurement and SEM image of the gel. **A**, Rheological measurement with the mode of dynamic time sweep for the sample containing 2.0 wt% of the protein and 1.0 wt% of the polymer (strain value = 1% and frequency value = 10 rad/s). The inserted is an optical image of the resulting hydrogel. **B**, An SEM image of the gel. The bar represents 10 µm.

We observed rapid formation of hydrogels upon mixing a protein solution and a polymer solution (Figure 2A) which showed an optical image of a gel containing 1.0 wt% of the polymer and 2.0 wt% of the protein. If fixing the concentration of the polymer to be 1.0 wt%, the minimum protein concentration needed for hydrogel formation was about 1.8 wt%. We then choose the concentration of the polymer and the protein of 1.0 and 2.0 wt%, respectively, to make hydrogels for further analysis and application (Figure 2, Figure S4 & Figure S5).

The hydrogelation process was monitored by a rheometer with the mode of dynamic time sweep. As shown in Figure 2A, a hydrogel formed rapidly within seconds upon mixing the protein and the polymer solutions. The elasticity (G′) of the gel increased slowly and reached a final value of about 80 Pa at 3,600 seconds time point. The small G′ value of the resulting gel indicated a weak gel, which was a characteristic of physical protein hydrogels[20], [21], [22].

We then obtained the morphology of micro-structures in a freeze-dried gel by scanning electron microscopy (SEM). As shown in Figure 2B, we observed a 3D porous structure in the sample, which was also frequently observed in other reported freeze-dried hydrogel samples. The pore size was about 1–2 µm and the pores connected with each other to form a 3D network (Figure 2B).

### 3D cell culture

**Figure 3:**
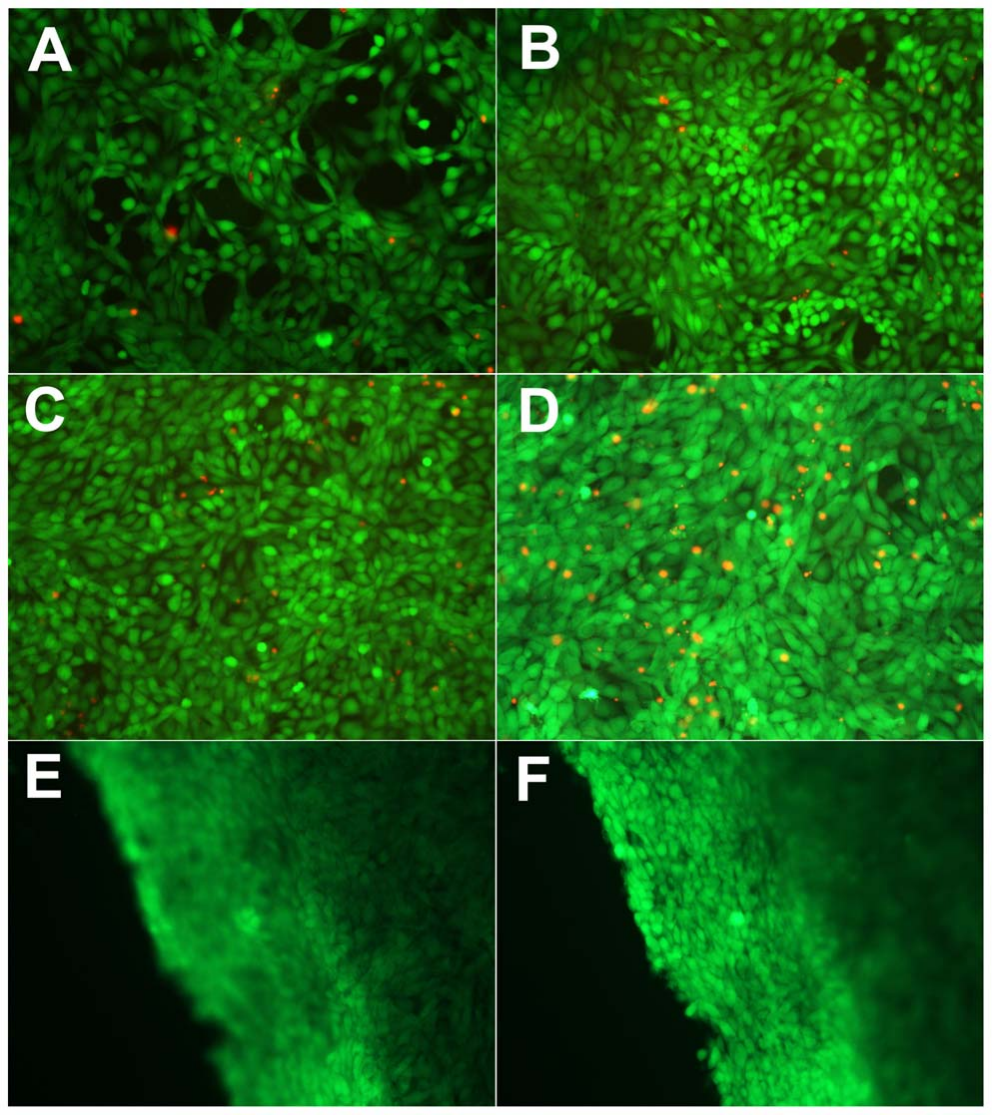
Live-dead assay of mMSC in hydrogels a continuous seven days in culture. A, day 1; B, day 3; C, day 5; D, day 7. The living cells were stained with calcein AM (green) and the dead cells were stained with EthD-1 (red). The images of E and F showed top and side views of a 3D cell-gel construct at day 7, respectively.

We then tested whether the hydrogels were suitable for 3D cells culture or not. The 3D cell-gel constructs were formed by mixing two solutions in 96-well plates, one phosphate buffer saline (PBS) solution containing 4.0 wt% of ULD-TIP-1 and the other Dulbecco's Modified Eagle's Medium (DMEM) solution containing 2.0 wt% of the polymer and mMSC supplemented with 10% FBS. After these components were well-mixed, the final density of cells was 2,000,000 cells/mL of gels (30 µL of gel/well). The results showed that mMSC grown very well in our ULD-TIP-1 protein-based hydrogel. Simultaneously, the mMSC could reach to a high cell density over a period of 7 days (Figure 3A–3D).

### Live-dead assay

**Figure 4:**
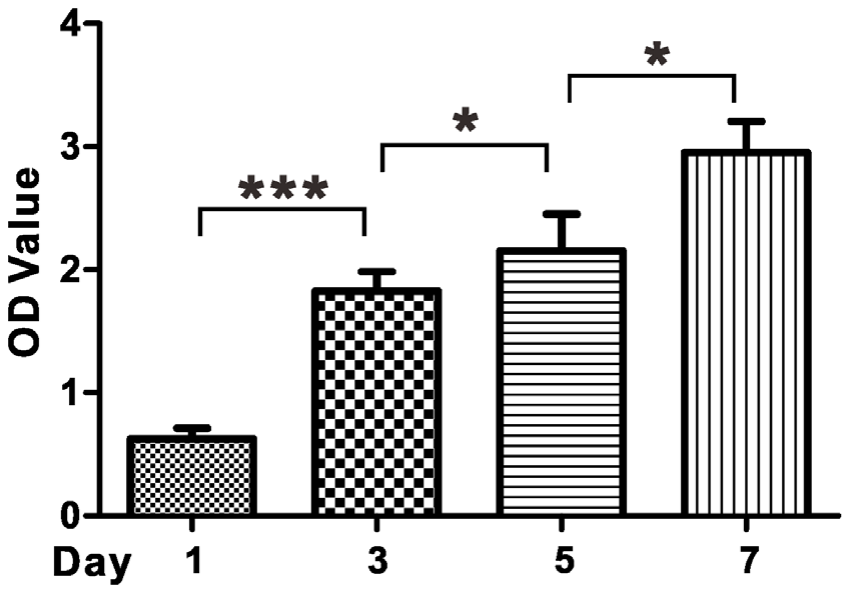
Cell proliferation rate of mMSC in gels determined by a CCK-8 assay. One asterisk (*) indicates p value smaller than 0.05 (p<0.05). Three asterisks (***) indicate p value smaller than 0.001 (p<0.001).

The live-dead assay was performed at different time points. As shown in Figure 3A–3D, mMSC adopted spindle or polyhedron shapes in 3D cell-gel constructs, which indirectly showed that the state of the mMSC were very good. Most of them were alive, as indicated by the cells in green color, and the dead cells were stained red (Figure 3A–3D). At the same time, the statistical data showed that cell survival rate was maintained at above 95% in the seven days of culture (Figure S7). The density of cells in cell-gel constructs kept increasing during the 7-day culture period (Figure 3, Figure 4). At day 7, we carefully took out a small piece of cell-gel construct, which was observed by the fluorescent microscopy. The top (Figure 3E) and side (Figure 3F) views of the cell-gel construct indicated that all cells were evenly distributed in it and both cells at the surface of and within the cell-gel construct were alive.

### CCK-8 assay

A CCK-8 assay was then used to determine the proliferation rate of mMSC in hydrogels. Figure 4 showed the results that the number of metabolic-active mMSC in hydrogels kept increasing during the 7-day culture period. The metabolic-active cell number at day 3, day 5, and day 7 was 184%, 230% and 359% more than that at day 1, respectively (Figure 4). Our physical hydrogels could be converted to homogeneous solution by firstly adding an equal volume of aqueous solution and then pipeting for several times[33]. Therefore, the mMSC post culture could be separated from cell-gel constructs and obtained after centrifugation from the homogeneous solutions. These results indicated that our protein-based hydrogel was suitable for 3D culture of mMSC, which might be used for *in vitro* large scale expansion of mMSC for tissue engineering and regenerative medicine.

## Conclusions

In summary, we have constructed a protein-based hydrogel system by a specific protein-peptide interaction. The simple mixing strategy for hydrogelation was convenient and biocompatible to cells encapsulation, which could guarantee its future applications in 3D cell culture and controlled delivery of pharmaceutical agents. We also demonstrated that our hydrogel system could provide excellent environments for mMSC. The mMSC kept dividing in hydrogels during 7-day culture period. The cells could be separated from cell-gel constructs post culture by a simple pipetting process. These results suggested its big potential for *in vitro* expansion of mMSC for further applications. We believed that our hydrogel system could also be used for the delivery of cells for cell therapy, which would be studied in our lab in near future.

## Supporting Information

Figure S1 Chemical structure of compound 1 (CGGGRGDGWRESAI).
(TIF)

Figure S2 1H NMR of compound 1. CGGGRGDGWRESAI: ^1^H NMR (300 MHz, DMSO-d_6_) δ 8.85–8.95 (t, 1H), 8.67–8.77 (t, 1H),8.15–8.35 (m, 6H), 7.88–8.14 (m, 7H), 7.55–7.61 (d, J = 7.58 Hz, 1H), 7.46–7.53 (m, 1H), 7.25–7.35 (d, 3H), 7.10 (s, 2H), 6.91–7.05 (m, 3H), 5.00 (s, 1H), 4.49–4.61 (m, 2H), 4.22–4.40 (m, 5H), 4.06–4.19 (m, 2H), 3.69–3.93 (m, 8H), 3.51–3.67 (m, 4H), 2.99–3.15 (m, 5H), 2.88–2.98 (m, 1H), 2.60–2.77 (m, 1H), 2.21–2.33 (m, 2H), 1.83–1.97 (m, 1H), 1.61–1.83 (m, 4H), 1.30–1.61 (m, 7H), 1.13–1.25 (m, 4H), 0.76–088 (t, 6H).
(TIF)

Figure S3 ^1^H NMR of PEG (above) and PEG-peptide (below). The hydrogen peak at 6.7 ppm disappeared completely, indicating the Michael addition reaction of maleimide with cysteine.
(TIF)

Figure S4 Rheological measurement with the mode of dynamic frequency sweep at the strain of 1% for the gel containing 1 wt% of PEG-peptide and 2 wt% of protein. The solution gradually became a hydrogel with the frenquency from low to high.
(TIF)

Figure S5 Rheological measurement with the mode of dynamic strain sweep at the frequency of 10 rad/s for the gel containing 1 wt% of PEG-peptide and 2 wt% of protein. The hydrogel showed weak stain dependences from 0.1% to 10%, with the G′ value of about 80 Pa.
(TIF)

Figure S6 The purified ULD-TIP1 protein was analyzed by 15% SDS-PAGE. M, Marker.
(TIF)

Figure S7 The survival rate of mMSC cells were cultured in the hydrogel at different day.
(TIF)
