# Supplementary figures and images for: A Protein-Based Hydrogel for In Vitro Expansion of Mesenchymal Stem Cells
Source: PLoS One. 2013 Sep 19;8(9):e75727. doi: 10.1371/journal.pone.0075727 (PMC3777955; doi:10.1371/journal.pone.0075727)

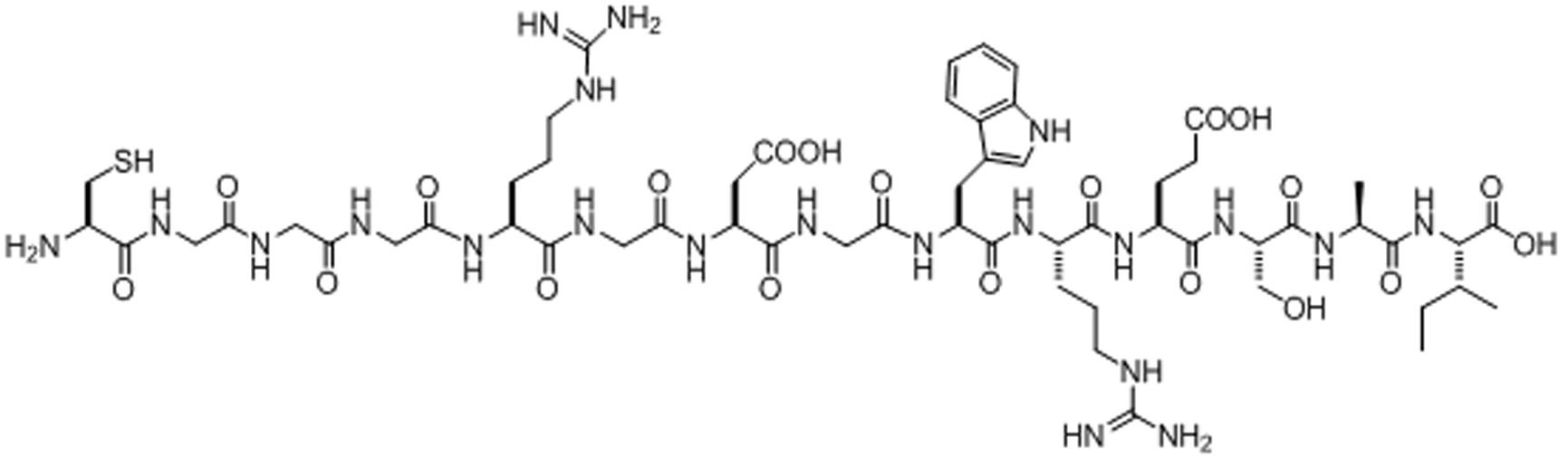

Supplement: Figure S1 — Chemical structure of compound 1 (CGGGRGDGWRESAI). (TIF) [file pone.0075727.s001.tif]

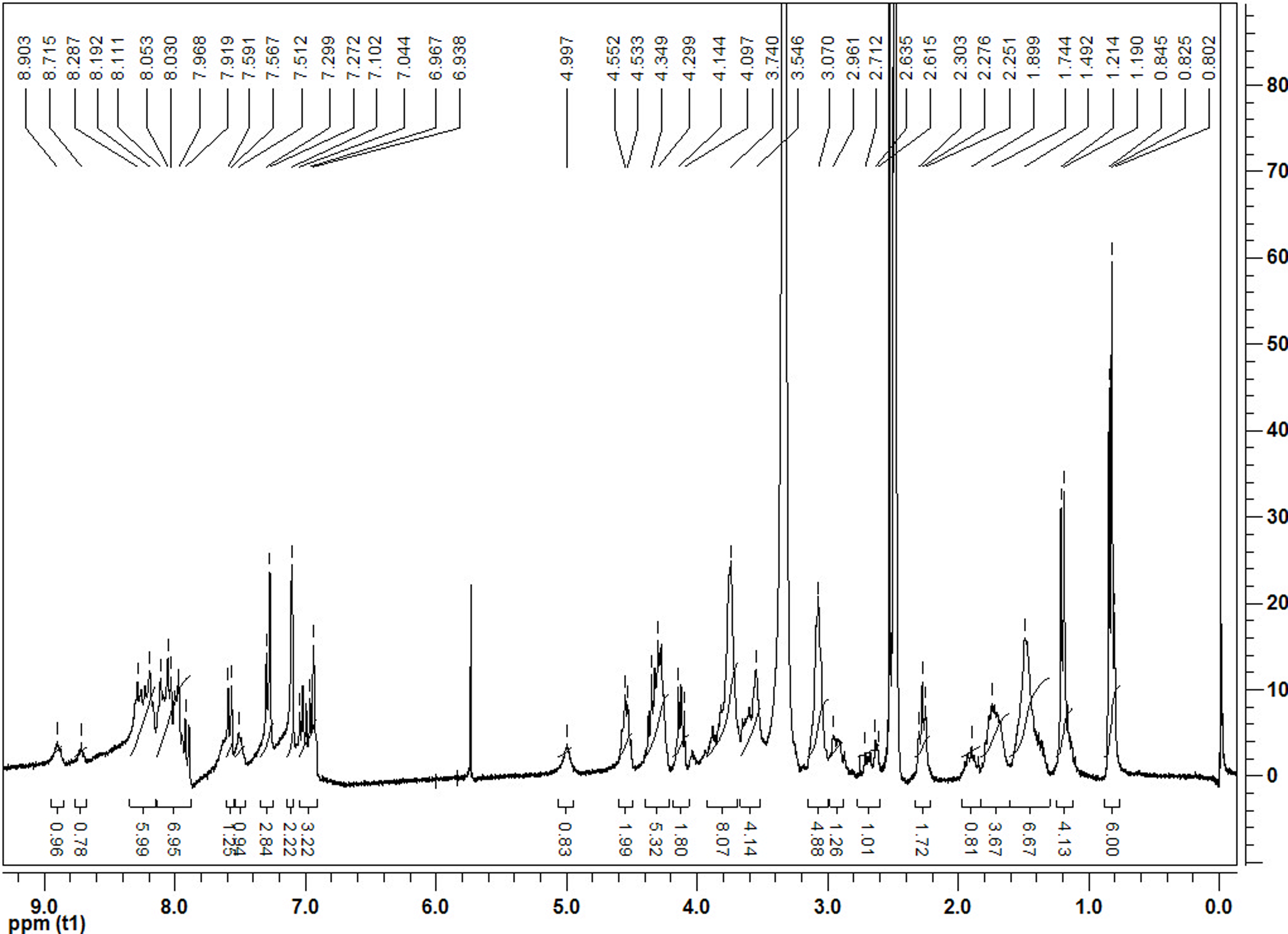

Supplement: Figure S2 — 1H NMR of compound 1. CGGGRGDGWRESAI: 1H NMR (300 MHz, DMSO-d6) δ 8.85–8.95 (t, 1H), 8.67–8.77 (t, 1H),8.15–8.35 (m, 6H), 7.88–8.14 (m, 7H), 7.55–7.61 (d, J = 7.58 Hz, 1H), 7.46–7.53 (m, 1H), 7.25–7.35 (d, 3H), 7.10 (s, 2H), 6.91–7.05 (m, 3H), 5.00 (s, 1H), 4.49–4.61 (m, 2H), 4.22–4.40 (m, 5H), 4.06–4.19 (m, 2H), 3.69–3.93 (m, 8H), 3.51–3.67 (m, 4H), 2.99–3.15 (m, 5H), 2.88–2.98 (m, 1H), 2.60–2.77 (m, 1H), 2.21–2.33 (m, 2H), 1.83–1.97 (m, 1H), 1.61–1.83 (m, 4H), 1.30–1.61 (m, 7H), 1.13–1.25 (m, 4H), 0.76–088 (t, 6H). (TIF) [file pone.0075727.s002.tif]

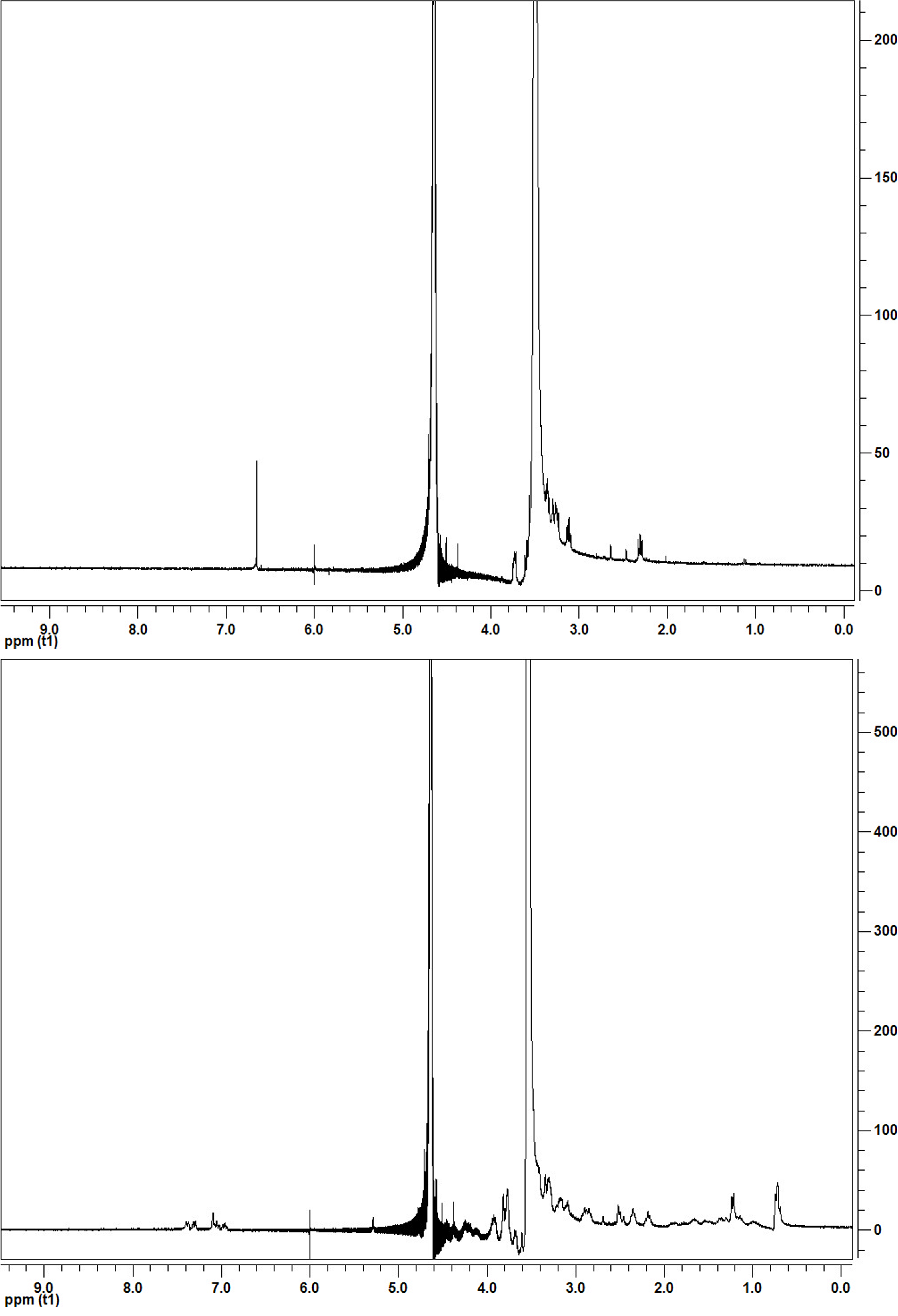

Supplement: Figure S3 — 1H NMR of PEG (above) and PEG-peptide (below). The hydrogen peak at 6.7 ppm disappeared completely, indicating the Michael addition reaction of maleimide with cysteine. (TIF) [file pone.0075727.s003.tif]

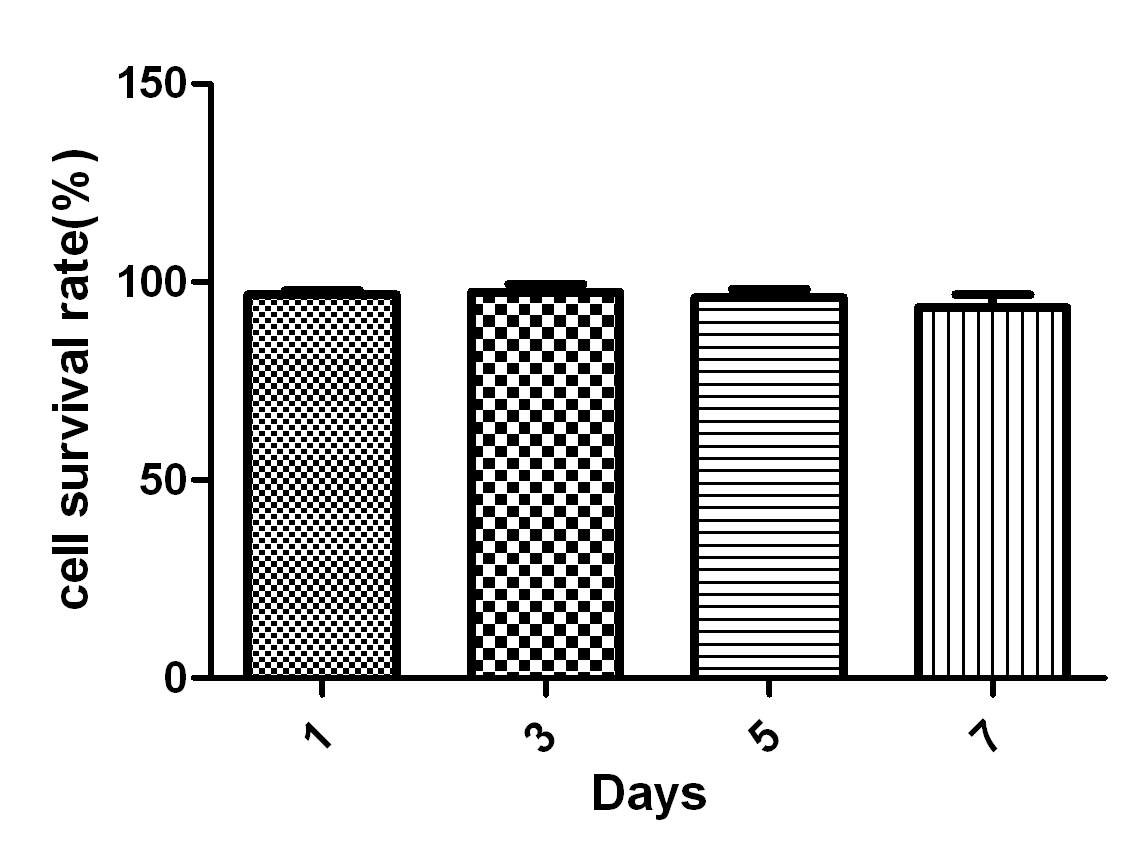

Supplement: Figure S7 — The survival rate of mMSC cells were cultured in the hydrogel at different day. (TIF) [file pone.0075727.s007.tif]
